# Sociodemographic and Clinical-pathological Study of Molecular Subtitles of Breast Carcinoma in a Reference Unit of Maranhão

**DOI:** 10.1055/s-0040-1719147

**Published:** 2020-12-21

**Authors:** Ana Paula Almeida Miranda Reis, Cecilma Miranda de Sousa Teixeira, Adriano Rêgo Lima de Medeiros, Karlla Zolinda Cantão Chaves, Camila Rosa de Albuquerque, Mateus Rufino Melo

**Affiliations:** 1 Centro de Ciências Sociais, Saúde e Tecnologia, Universidade Federal do Maranhão, Imperatriz, MA, Brazil

**Keywords:** breast neoplasms, immunohistochemistry, health profile, oncology, staging of neoplasms, neoplasias da mama, imunohistoquímica, perfil de saúde, oncologia, estadiamento de neoplasias

## Abstract

**Objective** To evaluate the distribution of the main sociodemographic and clinical-pathological characteristics in women with breast cancer according to the molecular profile by immunohistochemistry.

**Methods** A cross-sectional, retrospective, analytical and quantitative study was performed, with an analysis of 137 medical records from January 2015 to December 2018 of women attending the High Complexity in Oncology Unit of the city of Imperatriz, state of Maranhão, Brazil. The immunohistochemical profile of tumors based on the estrogen and progesterone receptor, Human Epidermal growth factor Receptor-type 2 (HER2) overexpression and Ki67 cell proliferation index was defined, from which six molecular subtypes were determined: luminal A, luminal B-HER2 negative, luminal B-HER2 positive, triple negative, overexpression of HER2 and inconclusive.

**Results** A total of 52.6% of the patients were postmenopausal, mean age 52.1 years old, brown (56.2%), had a schooling level < 9 years (40%), staging > IIB (52.6%) and 23.4% had metastasis. Invasive ductal carcinoma accounted for 84.7%, tumor size was 2 to 5 cm (48.9%), with lymph node involvement (56.2%), axillary lymphadenectomy in 67.2%, and mastectomy in 73.7% of the patients. The most frequent molecular subtype was the luminal B-HER2 negative (36.5%), and the luminal A subtype showed characteristics of better prognosis when compared with the others.

**Conclusion** It was concluded that in the association of molecular subtypes with sociodemographic and clinical-pathological characteristics, there were no statistically significant results obtained, except for complementary therapy, referring to hormone therapy, and there was a high index of metastasis at diagnosis, which was a worrying factor and indicative of failures in the screening and early diagnosis of this population.

## Resumo

**Objetivo** Avaliar a distribuição das principais características sociodemográficas e clínico-patológicas em mulheres com câncer de mama segundo o perfil molecular pela imunohistoquímica.

**Métodos** Estudo transversal, retrospectivo, analítico, descritivo e quantitativo, com análise de 137 prontuários do período de janeiro de 2015 a dezembro de 2018 de mulheres atendidas na Unidade de Assistência da Alta Complexidade em Oncologia da cidade de Imperatriz, MA, Brasil. Foi definido o perfil imunohistoquímico dos tumores baseado na avaliação dos receptores de estrogênio e progesterona, superexpressão de HER2 e índice de proliferação celular Ki67, de onde foram determinados seis subtipos moleculares: luminal A, luminal B-HER2 negativo, luminal B-HER2 positivo, triplo negativo, superexpressão de HER2 e inconclusivo.

**Resultados** Foi demonstrado que 52,6% das pacientes eram pós-menopausadas, com idade média de 52,1 anos, pardas (56,2%), tinham grau de escolaridade < 9 anos (40%), estadiamento > IIB (52,6%) e 23,4% tinham metástase. Carcinoma ductal invasivo representou 84,7%, o tamanho tumoral foi de 2 a 5 cm (48,9%), com comprometimento linfonodal (56,2%), com linfadenectomia axilar em 67,2% e mastectomia em 73,7% das pacientes. O subtipo molecular mais frequente foi o luminal B-HER2 negativo (36,5%), e o subtipo luminal A apresentou características de melhor prognóstico em relação aos demais.

**Conclusão** Concluiu-se que na associação dos subtipos moleculares com as características sociodemográficas e clínico-patológicas não se obteve resultados com significância estatística, exceto para terapia complementar, referente à hormonioterapia, e houve elevado índice de metástase ao diagnóstico, o que representou um fator preocupante e indicativo de falhas no rastreio e diagnóstico precoce dessa população.

## Introduction

Breast cancer or breast carcinoma is an important public health problem due to its high incidence and mortality, in addition to being the major cause of cancer death in women worldwide. 1 It is the most common malignant disease among women, with an estimated 1,67 million new cases diagnosed worldwide each year. 2 In Brazil, 59,700 new cases of breast cancer are estimated for each year of the biennium 2018–2019, with an estimated risk of 56.33 cases per 100,000 women. In the state of Maranhão, 720 new cases were estimated, 280 of which for the capital. 3

Malignant breast neoplasm is not a single disease, since it comprises many biologically distinct entities, with different pathological characteristics and clinical implications. 4 The tumor heterogeneity of this pathology is a major challenge to be faced, since tumors with the same histological types, stages and degrees of differentiation may have different prognostic outcomes and responses to the instituted treatment. 5 Accumulated evidence suggests that this presentation can be justified by the complexity of this neoplasm and the arsenal of molecular changes that lead to different responses to treatment, and should receive different therapeutic strategies. 4 5

Thus, the precise grouping of breast cancers into clinically relevant subtypes is of particular importance for therapeutic decision-making. 4 After studying several gene expression panels, five intrinsic breast carcinoma subtypes were identified that correlate with the prognosis: Luminal A, Luminal B Human Epidermal growth factor Receptor-type 2 (HER-2) negative, Luminal B HER-2 positive, HER2-enriched and triple-negative. Genomic evaluation is the gold standard for the classification of tumors in these subtypes, but it is complex and has a high cost, making it difficult to use in clinical practice. However, there is a great similarity between its outcomes and the ones provided by immunohistochemistry (IHC), through classic markers like estrogen receptor (ER), progesterone receptor (PR), and HER2. This makes IHC the most used exam for molecular classification, which in turn will guide the target therapies. 6 7

Nowadays, the expression of ER, PR, HER2 and Ki67 (monoclonal antibody that measures cell growth fraction) receptors, along with the variable clinicopathological data, such as nodal involvement, tumor size, type, grade of tumor and resection margins are commonly used to choose the treatment and predict the prognosis of the disease. 5 7

Considering the current implications in the therapeutic approach to breast cancer, the performance of studies that provide a better understanding of the distribution of the disease from its heterogeneities in women justifies the present research. Thereby, the objective is to evaluate the distribution of the main sociodemographic and clinical-pathological characteristics in women with breast cancer according to the molecular profile provided by IHC.

## Methods

Cross-sectional study, retrospective, quantitative, descriptive and analytical, whose population was formed by all women diagnosed with breast cancer performed between January 2015 and December 2018 ( *n* = 152), attended at the High Complexity Assistance Unit on Oncology (UNACON, in the Portuguese acronym), from the city of Imperatriz, state of Maranhão, Brazil. All medical records from patients who had ductal carcinoma in situ ( *n* = 2); lobular carcinoma in situ ( *n* = 3) and those with incomplete analysis of the immunohistochemical profile ( *n* = 10) were excluded; therefore, 137 records were selected for the present study.

Data recruitment was performed through an active search in the medical records of the oncology file of the aforementioned reference center, whose collection of information from patients took place according to a script with a standardized form. The definition of the immunohistochemical profile of the tumors was performed based on the results of reports issued by pathological anatomy services, based on the evaluation of ER and PR, HER2-enriched and Ki67 cell proliferation index. According to the results obtained, six immunohistochemical subgroups were defined: luminal A (RE + , RP + , HER2-), luminal B-HER2 negative (RE+ and/or RP + , HER2-), luminal B-HER2 positive (RE+ and/or RP + , HER2 + ), triple-negative or basal-like (RE-, RP-, HER2-), HER2-enriched (RE-, RP-, HER2 + ) and Inconclusive (HER2 inconclusive after IHC testing and fluorescent in situ hybridization [FISH]). According to the 2013 St. Gallen Consensus, the Ki67 index is considered as low or negative when < 14% and as positive or high when equal or higher than this value, 6 this being 14% the cutoff value used for the present study.

Sociodemographic variables were analyzed, such as date of diagnosis; age at diagnosis (in years), categorized as ≤ 50 and > 50 years old (cutoff point validated as a marker for menopausal status) 8 ; self-reported race; marital status; education level; place of residence; family history of cancer, smoking and drinking; and clinical-pathological characteristics: tumor size; histological type; lymphatic impairment; staging according to the TNM Classification of the American Joint Cancer Committee; presence of metastasis at diagnosis, type of surgery; axillary approach; and complementary therapy. The collected data were stored in a specific database created in Microsoft Excel version 2016 (Microsoft Corporation, Redmond, WA, USA). After checking for mistakes and inconsistencies, statistical analysis of the data was performed in IBM SPSS Statistics for Windows, Version 24.0 (IBM Corp., Armonk, NY, USA). 9 10

Initially, descriptive analyzes were performed using relative and absolute frequencies of sociodemographic, clinical and pathological characteristics. To assess possible associations between variables, the chi-squared and Fisher-Freeman-Halton tests were used. 11 All tests were performed at 5% significance.

Research approved by the Ethics and Research Committee through Plataforma Brasil under Statement n°. 3.213.056.

## Results

The analysis of the research results showed that the average age at diagnosis was 52.1 years old (standard deviation [SD] = 11.58). Considering the menopausal status, the study showed that 72 (52.6%) patients were diagnosed in the postmenopause; the predominant self-declared race was brown, with 77 patients (56.2%). As for the origin, 83 (60.6%) were from other cities in Maranhão, and Imperatriz had 51 patients (37.2%), and according to marital status, 85 (62%) were married or cohabited. Regarding schooling, 54 (40%) patients had < 9 years of schooling. As there is no information related to smoking, alcohol consumption (current or past) and family history of cancer in all medical records, these data were analyzed in 120 patients. Smoking was denied by 85 (70.8%) patients, alcohol consumption was denied in 79 (65.8%) patients, and the family history of cancer was positive in 62 (51.7%) women. The distribution of the main sociodemographic and clinical characteristics is described in Table 1 .

**Table 1:** Sociodemographic and clinical characteristics of patients

Variables		n	%
Age at diagnosis ( *n* = 137)	Average	52.1	–
	Standard deviation	11.8	–
Menopausal State	Premenopause	65	47.4
	Postmenopause	72	52.6
	TOTAL	137	100.0
Race	White	39	28.4
	Brown	77	56.2
	Black	2	1.4
	Yellow	19	14.0
	TOTAL	137	100.0
Regrouped Races	White	39	28.4
	Non-white	98	71.6
	TOTAL		
City	Imperatriz-MA	51	37.2
	Other cities in MA	83	60.6
	Other states	3	2.2
	TOTAL	137	100.0
Marital Status	Unmarried	32	23.4
	Married or Cohabitant	85	62.0
	Divorced	6	4.4
	Widowed	14	10.2
	TOTAL	137	100.0
Level of Schooling	Illiterate	19	14.1
	< 9 years	54	40.0
	9 to 12 years	43	31.9
	> 12 years	19	14.1
	TOTAL	135	100.0
Smoking	Yes (current or past)	35	29.2
	No	85	70.8
	TOTAL	120	100.0
Alcohol Consumption	Yes (current or past)	41	34.2
	No	79	65.8
	TOTAL	120	100.0
Family history of cancer	Yes	62	51.7
	No	58	48.3
	TOTAL	120	100.0

The pathological characteristics, as described in Table 2 , showed that the prevalent molecular subtype was luminal B- HER2 negative with 50 patients (36.5%). Regarding the type of surgery, 101 (73.7%) patients underwent mastectomy and, according to the histological type, 116 (84.7%) had invasive ductal carcinoma, 7 (5.1%) had another histological type, of which 5 (3.6%) were mucinous carcinoma, 1 (0.7%) invasive clear cell carcinoma and 1 (0.7%) invasive papillary carcinoma.

**Table 2:** Pathological profile of patients

Variables		n	%
Molecular Subtype	Luminal A	27	19,7
	Luminal B- HER2 negative	50	36.5
	Luminal B- HER2 positive	18	13.1
	Triple-negative	18	13.1
	HER2-enriched	17	12.4
	Inconclusive	7	5.1
	TOTAL	137	100.0
Surgery type	Mastectomy	101	73.7
	Breast-conserving	34	24.8
	Not performed	2	1.5
	TOTAL	137	100.0
Axillary approach	Sentinel node biopsy	20	14.6
	Axillary lymph node dissection	92	67.2
	Not performed	25	18.2
	TOTAL	137	100.0
Histological type	Invasive ductal carcinoma	116	84.7
	Invasive lobular carcinoma	14	10.2
	Others	7	5.1
	TOTAL	137	100.0
TNM staging	≤ II B	65	47.4
	> II B	72	52.6
	TOTAL	137	100.0
Tumor size	< 2 cm	35	25.5
	2 to 5 cm	67	48.9
	> 5 cm	35	25.5
	TOTAL	137	100.0
Lymph node impairment	Positive	77	56.2
	Negative	60	43.8
	TOTAL	137	100.0
Metastasis	Positive	32	23.4
	Negative	105	76.6
	TOTAL	137	100.0
Location of metastasis	Bones	17	43.6
	Lungs	15	38.5
	Liver	7	17.9
	TOTAL	39	100.0
Complementary therapy	Chemotherapy	89	45.6
	Radiation therapy	54	27.7
	Hormone therapy	47	24.1
	Not performed	5	2.6
	TOTAL	195	100.0

According to the TNM staging, 72 (52.6%) patients were diagnosed in stage > IIB (advanced). 9 Regarding the tumor size, there was a predominance of tumor between 2 to 5 cm in 67 (48.9%) patients. For lymph node impairment, 77 (56.2%) patients were positive, and metastatic disease at diagnosis was found in 32 women (23.4%), some with > 1 metastatic site involved, the most frequent being bone, with 17 cases (38.5%), pulmonary with 15 cases (38.5%) and hepatic with 7 cases (17.9%). Regarding complementary therapy, almost all patients received at least one type of approach, 89 (45.6%) for chemotherapy, 54 (27.7%) radiation therapy, 47 (24.1%) hormone therapy and 5 (2.6%) did not undergo any type of systemic therapy.

Also, regarding the treatment used, it is possible to highlight that 54 patients (39.4%) underwent breast surgery and radiotherapy, which consists of local treatment of the pathology.

Table 3 shows the distribution of the main clinical, sociodemographic and pathological characteristics according to the subtypes of breast cancer classified by IHC. However, in general, most of the variables studied predominated in the HER2 negative luminal B subtype. Even so, relevant results were found in the individual analysis of each molecular subtype in relation to most of the study variables. According to the menopausal status, the predominance of the negative luminal molecular subtype B-HER2 was found both among premenopausal women, with 25 patients (38.5%), and in postmenopausal women, with 25 (34.7%) patients.

**Table 3:** Sociodemographic, clinical and pathological profile in relation to the molecular subtype

	MOLECULAR SUBTYPE												
	LA		LB neg		LB pos		HER2-enr		TN		Inc		*p-value*
Age at diagnosis (menopausal status)													
≤ 50 years old	12	18.5	25	38.5	9	13.8	8	12.3	7	10.8	4	6.2	*0.96* *
> 50 years old	15	20.8	25	34.7	9	12.5	9	12.5	11	15.3	3	4.2	
Race													
White	9	23.7	11	28.9	6	15.8	6	15.8	3	7.9	3	7.9	*0.56* *
Non-white	18	18.2	39	39.4	12	12.1	11	11.1	15	15.2	4	4.0	
Family history of cancer													
Yes	13	21.0	26	41.9	8	12.9	10	16.1	4	6.5	1	1.6	*0.09* *
No	9	15.5	19	32.8	7	12.1	6	10.3	12	20.7	5	8.6	
TNM Staging													
≤ II B	18	27.7	24	36.9	7	10.8	6	9.2	6	9.2	4	6.2	*0.20* *
> II B	9	12.5	26	36.1	11	15.3	11	15.3	12	16.7	3	4.2	
Tumor size													
< 2 cm	12	34.3	11	31.4	4	11.4	2	5.7	4	11.4	2	5.7	*0.41* **
2 to 5 cm	10	14.9	25	37.3	8	11.9	12	17.9	8	11.9	4	6.0	
> 5 cm	5	14.3	14	40.0	6	17.1	3	8.6	6	17.1	1	2.9	
Lymph node impairment													
Positive	11	14.3	27	35.1	13	16.9	11	14.3	11	14.3	4	5.2	*0.38* *
Negative	16	26.7	23	38.3	5	8.3	6	10.0	7	11.7	3	5.0	
Metastasis													
Positive	4	12.5	12	37.5	4	12.5	5	15.6	7	21.9	0	0.0	*0.30* **
Negative	23	21.9	38	36.2	14	13.3	12	11.4	11	10.5	7	6.7	
Performed chemotherapy													
Yes	18	20.2	25	28.1	14	15.7	13	14.6	14	15.7	5	5.6	*0.14* **
No	9	18.8	25	52.1	4	8.3	4	8.3	4	8.3	2	4.2	
Performed radiation therapy													
Yes	12	22.2	23	42.6	8	14.8	4	7.4	6	11.1	1	1.9	*0.39* *
No	15	18.1	27	32.5	10	12.0	13	15.7	12	14.5	6	7.2	
Performed hormonotherapy													
Yes	12	25.5	26	55.3	4	8.5	1	2.1	1	2.1	3	6.4	*<* *0.001* **
No	15	16.7	24	26.7	14	15.6	16	17.8	17	18.9	4	4.4	

Abbreviations: HER2-enr, HER2-enriched; Inc, Inconclusive (Immunohistochemistry + FISH); LA, Luminal A; LB neg, Luminal B- HER2 negative; LB pos, Luminal B- HER2 positive; TN, Triple-negative.

* Chi-squared test.

** Fisher-Freeman-Halton test.

Regarding race, regardless of the molecular subtype, the prevalence was of non-white patients (brown, black, yellow and indigenous), however, the largest distribution of white patients was among the negative B-HER2 luminal tumors, with 11 (78%) tumors of this subtype. The family history of cancer was predominantly positive in most of the molecular subtypes of the study (luminal A, luminal B-HER2 negative, luminal B-HER2 positive and HER2-enriched).

When it comes to tumor size, most of the tumors > 5.0 cm and between 2 to 5 cm were found in luminal B-HER2 negative (40% and 37.3%, respectively); however, tumors < 2 cm were prevalent in the luminal subtype A, with 12 (34.3%) occurrences, which corresponded to 44.4% of the cases related to this subtype.

Regarding lymph node impairment, it is worth mentioning the positive character, which predominated in the negative subtype B-HER2 with 27 patients (35.1%). Specifically, in relation to the positive luminal B-HER2, 13 patients (72% of this subtype) presented lymph node positivity, and in relation to luminal A, 16 women (59.2% of this group) presented negative lymph node impairment.

Regarding staging, luminal subtype A deserves to be highlighted, in which most tumors presented in stages ≤ IIB, which corresponded to 18 patients (66.7% of this subtype), while for the negative triple subtype, 12 women (66.6% of this group) were > IIB at diagnosis.

Regarding metastasis, most patients who presented positive results of this characteristic at diagnosis belonged to the negative B-HER2 luminal subtype, which corresponded to 12 (37.5%) out of the 32 cases of metastasis recorded in the study. However, in the individual analysis of each subgroup, 7 out of the 18 patients belonging to the triple negative group showed metastasis at diagnosis, a total of 38.8%, which evidenced this subtype with the highest percentage of metastasis. Regarding the inconclusive subgroup, there were no cases of metastasis.

Also, it is possible to highlight that among patients with metastasis, 29 out of 32 (9.6%) underwent mastectomy, 2 (6.2%) conservative surgery, and 1 (3.1%) patient did not receive surgical treatment.

Regarding complementary therapy, the data showed that for chemotherapy, both the luminal groups B-HER2 positive and triple negative showed prevalence of this treatment, performed in 14 of the 18 cases (77.7%) of each subtype. Regarding radiation therapy, it was prevalent in the negative subtype B-HER2, with 23 out of the 54 (42.6%) patients undergoing radiation therapy. Regarding hormone therapy, the analysis of the relationship between this treatment and the molecular subtypes was statistically significant ( *p* < 0.001), demonstrating the prevalence of this modality in the negative B-HER2 luminal subtype, with 26 out of the 47 (55.3%) of the hormone therapy performed. On the other hand, in the positive B-HER2 luminal subtype, 14 out of 18 patients (77.7%) did not undergo hormonal therapy, demonstrating that only 4 (22.3%) out of 18 patients received this therapeutic modality.

## Discussion

Regarding age, our results are similar to the research that evaluated the profile of patients with breast cancer in Cuiabá, state of Mato Grosso, Brazil, in which the average age of the patients was 51.8 years old. 12 According to Magalhães et al., 13 this data corroborates with previous studies, which state that the risk of breast cancer increases with age, being relatively rare before the age of 35 years old.

Regarding smoking and alcohol consumption, the results obtained in the present study were similar to other studies performed in Belém, state of Pará, 13 and in São Paulo, state of São Paulo, 14 in which most patients had no history of smoking and alcohol consumption, as highlighted by the authors. These habits are modifiable risk factors, and smokers, ex-smokers and passive smokers are at increased risk of developing cancer, especially after menopause, and alcohol intake is a risk factor for all types of cancer, including breast cancer. 15

Regarding the level of schooling, the present study corroborates with research that evaluated the epidemiological profile of breast cancer in a reference hospital in the northern region, 14 where > 50% of the patients had not completed elementary and high school. However, it is noteworthy that the percentage of illiterate patients was 3.5% against 14.1% found in our study. This information is relevant in terms of the level of knowledge of the patients about the disease, its prevention and diagnosis, since this level is directly proportional to education. 14

Regarding marital status, in agreement with the studies by Farina et al., Llanos et al., and Haddad et al., 12 16 17 most women were married. Marital status is not considered a risk factor for the development of the disease, but the fact of having a partner is associated with better social support, optimism and quality of life among surviving women. 17

Considering the type of surgery, the results found in the present study showed that 73.7% of the patients underwent radical dissection, which is different from what was found in a study that evaluated the 10-year survival rate in patients with breast cancer in southeastern Brazil, where 53.6% of the women underwent conservative surgery. 18 Radical dissections are indicated in cases of invasive malignant tumors that occupy > 20% of the breast volume; 19 the high percentage of radical mastectomy performed in the present research may be justified by the prevalence of large tumors and in advanced stages of diagnosis.

When it comes to the axillary approach, a Spanish research that evaluated this approach in breast cancer for 20 years concluded that the percentage of axillary lymphadenectomy went from 91% at the beginning of the study to 34% at the end, which was attributed to the introduction of the sentinel lymph node biopsy and international criteria that modified the indication of axillary lymphadenectomy, avoiding morbidities associated with this procedure, especially lymphedema. 20 However, the results of the present study showed that 67.2% of women underwent axillary lymphadenectomy, in contrast to worldwide occurrences.

In the case of the histological type, the study of Pires et al. 21 corroborated with our results, in which 89.38% of the tumors were ductal invasive and 5.09% were lobular invasive; however, 0.66% of the patients had mucinous carcinoma, against 3.6% in the present study. It should be noted that invasive ductal carcinoma, in its pure form or in combination with other types, is the most common form of breast carcinoma reported in the literature. 21

According to the findings of the present research, for the immunohistochemical profile, the subtypes with the highest frequencies found were the luminal B-HER2 negative and the luminal A. These data corroborate with a large Brazilian multicenter study, which gathered 5,687 cancer cases across the country, whose objective was to identify the distribution of molecular subtypes in the 5 Brazilian regions, where there was the following variation in prevalence: luminal B, from 30.8% to 39.5%, luminal A from 24.1% to 28.8%, and triple negative, from 14% to 20.3%. The classifications adopted were similar to those of the present study in terms of the group of immunohistochemical markers, although there was a divergence between the positive B-HER2 luminal of this study and triple positive. 22

However, these results were different from international studies, in which they had a prevalence of luminal subtype A, with a frequency that corresponded to from 50 to 70% of cases, to the detriment of 19.7% in the present study. 7 23 24 25

Regarding menopausal status and correlation with the molecular subtype, our results differed from that observed by Cintra et al., 5 who in their study found that the triple negative subtype was prevalent in younger patients, whereas, in the present research, low frequency subtypes in premenopausal women were found in the analysis.

Considering race, the present study was different from other Brazilian studies, 5 26 which evaluated the profile of breast cancer patients, in which most of them were white. However, account should be taken of the miscegenation existing in the national population, 5 which justifies the presence of different results and the possibility of misclassification of the skin color in the present study, since the sample is composed of patients from northeastern Brazil, with a higher prevalence of blacks; 26 however, this group corresponded to 1.4% of the studied sample.

Considering the skin color and the subtype, a higher percentage of non-white skin color was demonstrated in the specific analysis, in the negative triple and a higher percentage of white skin color in the negative B-HER2 luminal. These data were similar to a North American study 27 that evaluated the factors that influence the stage of breast cancer diagnosis and showed a significant prevalence of triple negative in black patients and tumors with positive hormone receptor and negative HER2 in white patients.

The family history of breast cancer, especially when reported in first-degree relatives, is an important risk factor for the onset of the disease, considering that certain genetic alterations increase the risk of developing it. It is noted, however, that ∼ 9 out of 10 cases of this pathology occur among women without a family history. 28 In the present study, it was found that most patients had a positive family history and that among the molecular subtypes, the only one that did not show a prevalence of family history was the triple negative. However, it must be clarified that the data collected in the present study can be considered unreliable and/or ignored due to the search for information being restricted to consultation of hospital records.

Ganglion impairment has been recognized as an important prognostic factor in breast cancer, and the presence of positive axillary lymph nodes has been shown to predict increased risk of local and distant recurrence, with direct interference in mortality. 29 In the present study, high rates of lymph node impairment at diagnosis were observed, which corroborates with a bad prognosis in the studied population.

When it comes to tumor size, this is an important prognostic determinant, and together with the status of the axillary lymph node, are the two most important indicators in breast cancer. 21 With the advent of mammography and the greater awareness of the population of its importance, a significant reduction in the size of tumors at the time of diagnosis was observed in some developed countries; 30 however, approximately half of the patients evaluated in the present study had tumors between 2 and 5 cm at the time of diagnosis, which reflected a late diagnosis.

It is important to note that in a specific analysis of each subtype, luminal A showed a prevalence of small tumors and negative nodal involvement, which was compatible with what was described by Barreto-Neto et al. 26 by characterizing luminal subtype A as having the best prognosis, high survival rates and lower recurrence rates.

When it comes to stage at diagnosis, a cross-sectional study performed from 2000 to 2009 involving 87,969 Brazilian women 30 showed that there was a positive association between life in the poorest regions of the country (north, northeast and midwest) and late diagnosis of the disease. This same study demonstrated a gradual reduction in the rate of advanced breast cancer presentation, with a frequency of 59.5% in 2000 and of 50.9% in 2009. However, most patients in the present study were diagnosed in advanced stages of the disease, diverging from the evolutionary character observed in the country. Regarding metastasis at diagnosis, this was high among the population studied, especially when compared with other studies, with 23.4% of patients, while, nationally, percentages of 8.8%, 12.2% and 13.6% were found. 13 14 30 A study performed in a region that has one of the highest mortality rates in the world for breast cancer, sub-Saharan Africa, which brought together 2,558 cases of this tumor diagnosed from 2008 to 2015, showed that 18.4% of patients had metastatic disease in the diagnosis. 31 Advanced diagnosis stage shows greater difficulties and costs in treatment and is associated with greater morbidity and worse survival in high-income countries and in Brazil. Thus, a potential reason for the disproportionately high mortality from breast cancer in Brazil is late diagnosis. 32

It was observed that in the individual analysis of the molecular subtypes, in the triple negative and HER2-enriched, there were the highest rates of metastasis at diagnosis and, although it did not present statistical significance ( *p* = 0.30), this data is in agreement with other studies, 5 33 where these two subtypes were found among the most frequent metastatic disease.

In the case of complementary therapy, radiation therapy was more likely to be performed in patients with B-HER2 negative luminal breast cancer, in divergence from Cintra et al., 5 in which radiotherapy was more related to luminal subtype A. In this sense, Pires et al. 21 reported that radiation therapy has locoregional control of the disease as its main indication and its use is more common in the postoperative period to decrease the chances of recurrence; however, in some cases, it is used preoperatively to reduce tumor volume. Regarding chemotherapy, its adjuvant form is recommended for the subtypes HER2-enriched, luminal B-HER2 positive and triple negative, and may be prescribed or not for luminal HER2 negative tumors and not indicated for luminal A tumors. 6 The results of the present study corroborated this recommendation, since most of the chemotherapies performed were among the subtypes luminal B-HER2 positive, HER2-enriched and triple negative, although it should be noted that most of the patients with luminal A tumors received this therapy, which can be justified by the inclusion of neoadjuvant chemotherapies in the data collection of the present study.

In the case of hormone therapy, the presence of hormone receptors in tumor cells correlates with the benefit of hormone therapy, which uses substances that inhibit the activity of endogenous hormones progesterone and estrogen in the breast. 21 In agreement with these data, in the present study, the highest frequency of hormone therapy was between the subtypes luminal A and luminal B-HER2 negative, tumors that have hormone receptors. However, in the positive B-HER2 luminal category, despite containing these receptors, only 22.3% of the patients underwent hormone therapy ( *p* < 0.001), different from the pattern observed in a German study conducted from 2000 to 2016, 33 in which 67.8% of positive B-HER2 luminal patients received some type of hormone therapy, and the study conducted by Cintra et al., 5 which demonstrated that 76.7% of the patients belonging to this group received hormone therapy. Such divergence may be justified by the possibility of choosing neoadjuvant chemotherapy, initially, in tumors with some HER2 expression in the patients of the present study.

Regarding the inconclusive subtype, our results were compatible with the research 34 that investigated the clinical-pathological characteristics, treatment patterns and disease outcomes of tumors with doubtful HER2, where these tumors shared a greater similarity with HER2-negative disease. However, it is worth mentioning that the current consensus recommends the repetition of the HER2 test by FISH and/or IHC in equivocal cases, a fact that was demonstrated by the study of Xu et al., 35 where this recommendation proved that it could clarify ∼ 50% of doubtful cases; however, these tumors deserve further study, since there is limited information on the prognosis and the ideal treatment strategy for this population.

Based on these results, it is expected to contribute to the perception of the need to reinforce public health policies aimed at consolidating the national breast cancer screening program, especially for the group of women considered to be at higher risk and living in places with low health care, as they are susceptible to late diagnosis. It is also hoped to have demonstrated the importance of ensuring timely and appropriate treatment for women with breast cancer according to their molecular classification, and it is suggested that, based on the present research, new studies should be performed with a prospective approach with the intention of portraying the current situation in the region studied, considering the limitation of cross-sectional studies, specifically retrospective, as they do not demonstrate the reality of the moment.

## Conclusion

With the accomplishment of the present research, it was concluded that:

- The identification of women according to the molecular subtype by means of immunohistochemical classification was mainly in the negative subtype B-HER2.
- Sociodemographic characteristics came from women > 50 years old; brown race; from different cities than the city where the study was performed; married or cohabited; nonsmokers; nondrinkers; and with < 9 years of education; with a positive family history of cancer.
- The clinical-pathological characteristics were of mastectomized women, with axillary lymphadenectomy; carriers of invasive ductal carcinoma, staging > IIB (advanced); tumors from 2 to 5 cm; with lymph node impairment, without metastasis at diagnosis and chemotherapy as complementary therapy.
- That in the association of molecular subtypes with sociodemographic and clinical-pathological characteristics, no statistically significant results were obtained, except for complementary therapy, referring to hormone therapy. However, in the specific clinical analysis, it was observed that luminal subtype A had characteristics of better prognosis in relation to the other subtypes and that there was a failure in the implemented complementary therapy in relation to positive Luminal B-HER2, where the majority did not receive hormone therapy as recommended in the consensus.
- Although it is not part of the objectives of the present study, it was concluded that there was a high rate of metastasis at diagnosis, which is a worrying factor and indicative of failures in screening and early diagnosis of this population.
